# Surgical Reimplantation for the Correction of Vesicoureteral Reflux following Failed Endoscopic Injection

**DOI:** 10.1155/2011/352716

**Published:** 2011-01-09

**Authors:** Boris Chertin, Ksenia Prosolovich, Sagiv Aharon, Ofer Nativ, Sarel Halachmi

**Affiliations:** ^1^Department of Urology, Shaare Zedek Medical Center, the Faculty of Medicine Hebrew University, Jerusalem, 91031, P.O. Box 3235, Israel; ^2^Department of Urology, Bnai Zion Medical Center and the Faculty of Medicine, Technion Israel Institute of Technolog, 47 Golomb Street, Haifa 31048, Israel

## Abstract

*Purpose*. In recent years, endoscopic injection became the procedure of choice for the correction of vesicoureteral reflux in the majority of the centers. Unfortunately, endoscopic treatment is not always successful and sometimes requires more than one trial to achieve similar results to that of an open reimplantation surgery. Our aim of this study is to evaluate the feasibility and success rate of open ureteral reimplantation following failed endoscopic procedure. *Patients and Methods*. During 2004–2010, we evaluated 16 patients with persistent vesicoureteral reflux (grades II–IV) following failed endoscopic treatment. All patients underwent open ureteral reimplantation. All patients were followed with an ultrasound 6 weeks following surgery and every 6 months thereafter for an average of 22 months. Voiding cystography was performed at 3 months after surgery. *Results*. During unilateral open ureteral reimplantation, the implanted deposit from previous procedures was either excised, drained, or incorporated into the neotunnel with the ureter. Vesicoureteral reflux was resolved in all patients with 100% success rate. No new hydronephrosis or signs of obstruction developed in any of the patients. qDMSA renal scan was available in 8 patients showing improvement of function in 5 and stable function in 3, and no new scars were identified. *Conclusions*. Open ureteral reimplantation is an excellent choice for the correction of failed endoscopic treatment in children with vesicoureteral reflux.

## 1. Introduction

Vesicoureteral reflux (VUR) is a common occurrence in the pediatric age group resulting in potentially dangerous morbidities. In some patients, VUR if left untreated could result in renal scarring [1], hypertension, and end-stage kidney failure [2, 3]. In most instances, patients are managed conservatively by observation or medically with prophylactic antibiotics. For those patients who require interventional approach, the focus is given to selecting the best corrective endoscopic or surgical option. Since its introductions in 1984, many urological departments prefer endoscopic tissue-augmenting material injection procedure for the correction of VUR [4]. Subureteral injection of dextranomer/hyaluronic acid copolymer (Deflux) [5] material has been favored over other injectable agents because of its safety and efficacy [6, 7]. However, as more long-term follow-up studies emerge, the results indicate that endoscopic treatment has a lower success rate and a higher recurrence than open ureteral reimplantation surgery. Some authors do not recommended to proceed with Deflux treatment, after the procedure has failed in several attempts, due to lower success rate with each consequent injection [8]. In the setting of a failed endoscopic injection, both intravesical and extravesical reimplantation (IUR and EUR) surgical approaches are used [9, 10]. We report our experience with open ureteral reimplantation (OUR) for the correction of unsuccessful endoscopic implant and our evaluation of its viability and success rate when compared to other minimally invasive procedures.

## 2. Patients and Methods

During 2004–2008, 16 patients with persistent VUR following failed endoscopic treatment underwent open reimplantation and were followed until 2010. During this period, we performed around 400 endoscopic injections. Demographic data is summarized in Table 1. There were 8 boys and 8 girls, the mean age was 3.9 years (range: 2–6 years). In this group, 1 patient had 3 previous endoscopic injections, 7 patients had 2, and 8 patients had one injection. Mean VUR grade at presentation and before surgery was III (range II–IV).

Indications for operative treatment rather than conservative approach were absolute in all patients, including breakthrough infections, new scar development, global renal function deterioration of the refluxing kidney in two consecutive qDMSA (quantitative DMSA—method used at Bnai Zion) scans, and an additional male patient who had persistent reflux to a single functioning kidney.

All patients were evaluated by physical examination, renal ultrasound, voiding cystourethrogram (VCUG), and qDMSA scan prior to intervention. All had primary VUR and no other anatomical malformation or neurological impairment. In all patients either voiding dysfunction was ruled out or behavioral modification was encouraged prior to surgery.

The reasons to abort additional endoscopic injection and to proceed with open correction of VUR were single functioning kidney, deterioration of renal function in two consecutive qDMSA scans, laterally located ureteral orifices with a short intramural tunnel limiting the deposition of the Deflux material correctly, parental preferences as well as wide ureteral orifice diameter, which could not be managed with normal amount of bulking material.

Patients underwent reimplantation according to surgeon's preferences; the various methods used are listed in Table 1. These surgical procedures are already described extensively in the medical literature [11]. In 9 patients the Deflux deposit was excised from the ureter, in 4 patients it was incorporated into the neo-tunnel, and in 3 patients it was drained using a blade to open the bulk capsule and perform a suction of its content.

All patients were followed with an ultrasound at 6 weeks following surgery and every 6 months thereafter for an average of 22 months followup (range 8–36 months). VCUG was performed at 3 months following surgery and a qDMSA scan around one year after surgery. In all patients, we emphasized the need for bladder retraining and the avoidance of voiding dysfunction postoperatively.

## 3. Results

All surgeries were performed successfully with no intraoperative or postoperative complications. All patients voided spontaneously following catheter removal. Following discharge, one male patient developed transient fever without a clear source and was treated conservatively. Six weeks after surgery, sonographic ultrasound did not demonstrate any signs of new hydronephrosis; in 6 patients, the postvoid residual scan was included showing complete emptying of the bladder. VCUG performed in all patients three months after the operation showed complete resolution of the VUR. qDMSA scan available in 8 patients showed improved function in 5 and stable function in 3. No new scars were detected in the follow-up scans. In a mean followup of 4 years (2–6 years), all patients were developing well with serial renal ultrasounds showing appropriate renal growth in all patients.

## 4. Discussion

VUR is not uncommon in childhood with a reported frequency of around 1% [12]. Available treatment options depend both on the clinical presentation and the grade of VUR as set forth by the International Reflux Study Classification [13]. VCUG is the gold standard in making the diagnosis [14]. Renal function, clinical presentation, and VUR grade are essential variables for choosing whether a conservative or more invasive treatment is the preferred option for the management of the relevant case. 

As minimally invasive procedures become more readily available, many patients and their parents are finding them more appealing and influential during treatment selection [15, 16]. From the physician's view, it is important to maintain high success rate and provide excellent patient care, while simultaneously keeping the cost of health care low. This approach has influenced doctors to perform more procedures on an outpatient basis. Such factors have further contributed to the replacement of open surgery for endoscopic treatment. 

The main advantages of endoscopic treatment for VUR management include decreased posttreatment pain, bladder spasm, infection, and absence of surgical scar, especially in the setting of low grade VUR. The availability of this procedure in the outpatient setting, short procedure time, quick time to discharge, and minimal use of postoperative analgesics have shown to be beneficial for both the patient and the physician [17]. The ability to repeat this procedure after initial failure either with implantation or surgery is also an advantage [10].

More long-term follow-up studies have been published recently with success rates ranging in the low 80% after one treatment with Deflux, and reaching at best 98% with a second repeated procedure [18]. Thus, between 10%–20% of the patients, especially those with VUR grade of III and higher, will require repeated injections following one treatment with endoscopic implantation [19, 20]. While reflux resolution rates, with the use of Deflux, during early postoperative followup may seem reasonable (65.9%–80.2%), data warns of a 1-year total success rate including initial postoperative failure of 46.1% [21]. The Hydrodistention Implantation Technique (HIT) has offered patients up to 90% success rate for primary VUR as well as for correction of repeat endoscopic injection [22]. Nevertheless, both of the aforementioned procedures still leave a number of patients that will necessitate repeated corrective procedures. This translates into additional stress on the patient and the family, further office visits, repeated VCUG, more invasive procedures, and higher costs endured in order to correct the failed treatment. Endoscopic procedure seems promising with regards to cost, but careful evaluation of all expenses associated with this procedure has shown the opposite, that is, the cost for endoscopic subureteral injection exceeds that of outpatient open reimplantation for unilateral vesicoureteral reflux [23, 24]. 

When Deflux fails, after the first injection, patients are offered reinjection of Deflux or in the case of multiple failures: surgery. In the open surgery offered for reflux, both IUR and EUR play a crucial role in achieving close to 100% resolution of VUR. In recent years, both procedures were modified to be relatively rapid operations, not always requiring for the bladder to be opened during the surgery, ureteral tubing, and for many it is not necessary to leave a urethral catheter postoperatively. As these techniques are tailored and advanced, less morbidities such as bladder spasm, hematuria, and pain, in addition to less use of postprocedural analgesics are being reported [25–28]. In the past decade, EUR has become available in the outpatient settings, thus being comparable to endoscopic treatment [29]. Both procedures are reported to have relatively short postoperative stay of less than 24 hours (even in the inpatient settings) [30, 31]. When choosing the procedure of choice for patients with primary VUR, the hospital at which a patient receives treatment is the one of the most important features that ensure the selection [32]. While not every hospital has the ability to perform endoscopic procedure, most if not all can perform OUR with a high success rate. Furthermore, evaluation of corrective procedures offered for VUR after one treatment demonstrates resolution rate of 98%, 89%, and 78.5% success rates for OUR, HIT, and endoscopic implant, respectively [8, 22, 23]. These features make OUR more attractive as the procedure of choice for correction of failed Deflux in previous failed trials. We think it is further justified to offer OUR for patients who need maximal success rate including patients with new scars, and with deteriorating renal function in whom injection failed.

There is a debate whether a routine VCUG is needed following surgical reimplantation. Falkensammer et al. [33] retrospectively analyzed results of postoperative VCUG in 126 patients and found that it changed the management following surgery, in only 2 postoperative studies. The authors advocated performing VCUG only in patients with pyelonephritis. We think that for our patients who developed new scars or had deterioration of renal function prior to surgery, every effort should be made to verify treatment success or failure. In the study mentioned above, 2 patients developed pyelonephritis postoperatively. This infection can be devastating for the kidney in young children, especially those with a previously affected kidney and in patients with a single kidney. Hence, proper knowledge about residual or de-novo VUR would enable the physician to prevent it. Since we decided to give the maximal successful treatment option to each patient and given that not a lot of data has been published in the literature about after failed injection reimplantation, performing VCUG was justified in our study group.

We performed OUR in an inpatient setting as the cost of outpatient day surgery is similar to 48 hours hospitalization in our health care system. In both intravesical and extravesical approaches, the Deflux deposit was excised, drained, or incorporated into the neo-tunnel, allowing for minimal scaring and potential complications for the patient and technical ease for the physician. Keeping the deflux remnant occured only during extravesical reimplantation; in these cases the surgeon felt that removing the remnant may compromise the ureter, hence a meticulously tailored made tunnel capable to include the ureter and the remnant without any signs of mechanical obstruction was performed. Fixation of the distal ureter with a U shape suture to the caudal end of the neo-tunnel actually positioned the remnant in the designated place like following a perfect injection. If the surgeon was not satisfied with the results, excision and either dismembered or intravesical reimplantation were performed. These patients were constantly followed with serial ultrasound and none showed any new hydronephrosis. 

Even as an inpatient, all patients were discharged within 24–48 hours after EUR. We report 100% long-term success rate following the correctional repair of failed Deflux with minimal morbidity. Our results are comparative to previous report.

## 5. Conclusions

Both intravesical and extravesical techniques are an excellent choice for the correction of failed endoscopic treatment. The deflux deposit should not alter the surgeon preferable approach to reimplantation. If the Deflux deposit is adhered to the tissue, there is a possibility to excise, drain or, incorporate it into the neo-tunnel (in EUR) of the ureter without further complications. Whenever needed, an immediate high long-term success rate operation following failed endoscopic injection open reimplantation, regardless approach, is justified offering a very high success rate and a low complication rate.

## Figures and Tables

**Table 1:** Patient's characteristics.

No.	Sex	Age	Side	Degree of VUR	No. of previous injections	Deflux globe	Type of reimplant
1	M	5	RT	2	1	Incorporated	EV
2	M	4	RT	3	2	Excised	EV
3	M	3^2/12^	LT	2	2	Excised	EV
4	M	3^8/12^	LT	4	1	Incorporated	EV
5	F	2	LT	3	1	Excised	EV
6	F	2^6/12^	RT	3	2	Incorporated	EV
7	F	4	LT	2	2	Drained	EV
8	M	4	BL	3, 4	2	Excised	PL
9	M	6	BL	4, 4	3	Excised	PL
10	M	4	RT	4	1	Excised	PL
11	F	4	BL	2, 3	1	Drained	EV
12	F	3	LT	2	2	Drained	EV
13	F	4	BL	3, 3	2	Excised	Cohen
14	M	5	RT	4	1	Incorporated	EV
15	F	3	LT	3	1	Excised	EV
16	F	6	BL	2, 3	1	Excised	PL

EV: extra vesical

PL: politano leadbetter
